# Comparative Study on the Prokinetic Effects of Ginger, Caraway, and Peppermint in Relieving Gastrointestinal Disturbances in Pulmonary TB Patients: A Clinical Trial

**DOI:** 10.1002/fsn3.70338

**Published:** 2025-05-25

**Authors:** Asma Latif, Hajra Ahmad, Imran Bashir

**Affiliations:** ^1^ Department of Nutritional Sciences and Environmental Design, Faculty of Sciences Allama Iqbal Open University Islamabad Pakistan; ^2^ Sheikh Zayed Medical College and Hospital Rahim Yar Khan Pakistan

**Keywords:** anti‐tuberculosis treatment, gastrointestinal disturbances, prokinetic effect

## Abstract

Anti‐tuberculosis treatment (ATT) leads to adverse gastrointestinal disturbances (vomiting, nausea, postprandial distress) due to its prolonged duration in TB patients. This study assessed the prokinetic effects of ginger, caraway, and peppermint nutraceuticals in alleviating these symptoms in pulmonary TB patients on ATT. The selected nutraceuticals were procured, cleaned, dried, and crushed to perform proximate analysis, TPC, TFC, DPPH, ABTS, and FRAP. Further, a randomized clinical trial (Registered No. NCT06157034) involved 200 participants divided into four groups (placebo = 48, ginger = 47, caraway = 48, peppermint = 46), with 11 dropouts. Participants received varied dosages of selected nutraceuticals (Ginger: 1 g, Caraway: 2 g, and Peppermint: 3 mL daily), and data were collected at baseline, intervention (after 3 months), and washout (after 1 month) phases. Ginger and peppermint showed the highest moisture content (81.67%, 81.74%), while caraway had the highest protein (29.72%) and crude fiber (45.11%). Peppermint had the highest crude fat (54.47%) and crude fiber (4.81%). Moreover, ginger had the highest TPC (1035.51 mgGAE/g) and TFC (465.34 mgQE/g), with notable DPPH, FRAP, and ABTS values. Participants had a mean age (39.03 years), family income (17,385 ± 528.72Rs), and BMI (19.43 ± 5.67). Sputum testing and other biochemical analyses were also done. Energy and macronutrient consumption did not differ significantly across all phases (*p* > 0.05). The hierarchical heatmap graph depicted the intensity/severity of relieving gastrointestinal disturbances with the consumption of nutraceuticals. Thus, the results concluded that both ginger and peppermint groups, particularly during the washout phase, showed a promising reduction in GI disturbances, suggesting their potential as adjunctive therapy for TB patients on ATT.

## Graphical abstract

Selected nutraceuticals, rich in bioactive compounds, contributed to their potential prokinetic effect. Heatmap was employed to compare the intensities of GI disturbances among participants on the intervention of selected nutraceuticals in different phases. All three nutraceuticals significantly alleviated the GI disturbances in TB patients.

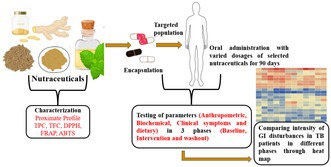

## 1 Introduction

Tuberculosis (TB) is a highly contagious disease that poses a significant threat to public health, leading to numerous fatalities, specifically a major concern in Pakistan and worldwide (Chumpitazi et al. 2018). Pulmonary tuberculosis (PTB) is a bacterial infection ( *Mycobacterium tuberculosis* ) that primarily affects the lungs but can also impact other organs, including the gastrointestinal (GI) tract (Mantilla et al. 2023; Zeng et al. 2023). The statistical data confirm that extra‐pulmonary tuberculosis (EPTB) signifies approximately 12% of all tuberculosis cases, with GI tuberculosis accounting for 11%–16% (Ammari et al. 2022). According to a WHO report (2022), Pakistan bears the highest TB burden in its region, accounting for 70% of the total incidence (Khursheed et al. 2024).

Anti‐tuberculosis treatment (ATT) is a standardized therapy used to treat TB infections (Kumar et al. 2018). The treatment of this disease is long enough, so there are many chances of adverse drug reactions in the gut of TB patients. However, GI disturbances are common among PTB patients, and about 6%–38% of patients with GI tuberculosis may present with concurrent PTB (Litvinjenko et al. 2023). GI disturbances can become apparent as symptoms such as loss of appetite, weight loss, nausea, vomiting, diarrhea, abdominal distention, abdominal pain, constipation, straining, urgency, anorexia, feeling of fullness, reflux, dyspepsia, and regurgitation (Modi et al. 2018). The occurrence of symptoms of GI disturbances before and during treatment is strongly linked with the previous discontinuation of therapy (Khan 2017; Modi et al. 2018). A case report described by Djaharuddin et al. (2020) reported the presenting complaint of abdominal pain during the treatment of intestinal TB among patients. In another study, the adverse GI disturbances during TB treatment were documented, with a 33% incidence of vomiting and nausea (Khan et al. 2022). If it is an on‐time‐based diagnosis, it can be curable with a 6‐month first‐line antibiotic and fewer GI complications (Tahiri et al. 2023). The prolonged and extensive use of ATT medications requires the exploration of alternative approaches to alleviate the severe repercussions of GI disturbances. In response to these challenges, the paradigm is shifting from exclusive reliance on allopathic medication to embracing Medical Nutrition Therapy (MNT) as a complementary therapeutic strategy (Lange et al. 2018).

Nutraceuticals, the bioactive compounds, have been considered safe for their potential prokinetic effects on managing GI disturbances in various conditions, including PTB (Bethapudi et al. 2022). However, prokinetics are substances that promote the movement of food through the GI tract and help improve digestion and absorption (Sakakibara 2021). Ginger, derived from the rhizome of *Zingiber officinale* , is a spice with a long history of use in traditional medicine, particularly known for its potential prokinetic effects to mitigate various gastrointestinal (GI) issues (Singh et al. 2022). Ginger has shown the ability to stimulate gastric emptying, enhance intestinal transit, and mitigate symptoms like nausea and vomiting (Kumari et al. 2020). Incorporating ginger into the diet makes it easily accessible and adaptable to individual preferences (Jamal 2023). The phytoconstituents of ginger proved helpful in mitigating infectious diseases with few adverse effects, such as blood coagulation and platelet aggregation (Chai et al. 2011). Bhaskar et al. (2020) showed a beneficial effect of ginger when given to patients in combination with ATT. Another scientific study proved the synergistic effect of ginger and ATT in relieving GI disturbance in TB patients (Kumar et al. 2017). Further, caraway seeds ( *Carum Carvi* ) have also been recognized for their potential to alleviate digestive discomfort (Javed et al. 2020). Caraway seeds and oil have gained popularity for their carminative properties, which suggests their ability to reduce gas and bloating (András et al. 2015). An in vivo RCT study reported no toxicity of caraway in rats by oral intake and proved safe (Auti and Kulkarni 2019). Scientific literature also supported the favorable ATT in combination with *C. carvi* by enhancing and modifying ATT kinetics among TB patients (Choudhary et al. 2014).

In recent times, scientific research has begun to explain the potential health benefits of peppermint, particularly in managing gastrointestinal (GI) disturbances (Shams et al. 2015). Peppermint oil acts as a prokinetic to enhance gut motility, aiding in the movement of food and reducing symptoms associated with GI disorders (Shams et al. 2015; Zhao et al. 2022). The phytoconstituents of peppermint demonstrated a favorable safety profile, with mild adverse effects such as heartburn (Ingrosso et al. 2022). Various studies investigating the effects of peppermint oil have primarily focused on its ability to alleviate GI disturbances in a variety of conditions (Currò et al. 2017; Shams et al. 2015; Thumann et al. 2019). The findings of an in vivo study proved peppermint to be a valuable natural remedy for treating ATT complications (Abdel‐Hameed et al. 2024). However, despite the therapeutic effect of nutraceuticals, there is limited scientific evidence directly addressing the prokinetic effects on gastrointestinal motility during ATT, especially in the context of managing symptoms associated with PTB. Therefore, the selection of ginger, caraway, and peppermint for this study was based on their well‐documented bioactive compounds and traditional use in managing GI disturbances. The current study aimed to procure and prepare ginger, caraway, and peppermint to characterize their proximate, TPC, TFC, and antioxidant profiles. Further, the resultant was supplemented to determine the prokinetic effect of these nutraceuticals with different dosages on GI disturbances among pulmonary tuberculosis patients.

## 2 Materials and Methods

The current study was divided into two parts. In the first part, the selected nutraceuticals (Ginger; *Zingiber officinale* , Caraway; *Carum carvi*, Peppermint; *Mentha spicata L*.) were processed to analyze the proximate, TPC, TFC, and antioxidant profiles. In the second part of the study, the resultant sample of each nutraceutical was encapsulated and administered to assess its prokinetic effect on managing gastrointestinal disturbances among pulmonary tuberculosis patients.

### 2.1 Procurement and Preparation of Sample

Samples of selected nutraceuticals were acquired from the local market in Rahim Yar Khan, Pakistan. The obtained samples were washed to remove dust. For ginger extraction, the method described by Umeh et al. (2013) was followed with little modification. The skin of the cleaned ginger sample was peeled, cut into smaller pieces, and sun‐dried. The dried samples were crushed using a manual blender (Braun, Romania). The resulting sample was stored in zipped bags. Subsequently, 200 g of the powdered ginger was macerated in 3 L of 80% ethanol for 72 h, with intermittent stirring to facilitate extraction. The mixture was then sieved through cheesecloth and cotton wool to obtain a clear filtrate. The resulting filtrate was concentrated into a semi‐solid extract in a vacuum chamber using a rotary evaporator (Samota et al. 2024). Further, the caraway seeds were cleaned, ground using a manual blender (Braun, Romania), and stored for further analysis. In addition, 60 g of sample was treated with 300 mL of 80% ethanol for 72 h. After extraction, the solvent was filtered and evaporated using a rotary evaporator (Model, YR02306, China) (Alara et al. 2021). Peppermint oil was prepared by using the method described by Siddeeg et al. (2018) with little modifications. The peppermint leaves were thoroughly rinsed with water and gently dried on paper towels. They were then crushed using a manual blender (Braun, Romania). The collected sample was then strained with a muslin cloth to collect the processed sample. The final product was then stored for further analysis. The obtained sample (30 g) was further treated with ethyl alcohol (350 mL) using a shake at 70°C for 6 h. The obtained solvent was removed under a laboratory fume hood at 37°C for 30 min. The final product was then refrigerated in a dark color bottle for further analysis.

### 2.2 Characterization of Selected Nutraceuticals

#### 2.2.1 Proximate Analysis

The proximate profile (moisture, ash, crude protein, fat, and fiber) of selected nutraceuticals was measured by the AOAC, 2000 method. Further, the carbohydrate content was determined by subtracting the difference from 100.

$$\begin{array}{c} \text{Carbohydrate}\;\left(\%\right)=100-\left(\text{Moisture}+\mathrm{Ash}+\text{Crude Protein}\right.\\ \;\left.+\text{Crude}\;\mathrm{Fat}+\text{Crude Fiber}\right)\% \end{array}$$

#### 2.2.2 Determination of Total Phenolic Content

The total phenolic content (TPC) in each resultant sample was determined using the Folin–Ciocalteu method (Lamuela‐Raventós 2018). Extract (50 μL) was mixed with distilled water (575 μL) and the Folin–Ciocalteu Reagent (125 μL) in a 1:16 ratio. After 5 min, 7% sodium carbonate (NaCO_3_) solution (1250 μL) was added to the treated sample. Further, the volume was adjusted to 3 mL with distilled water. The absorbance reading was noted at 760 nm using a UV–Vis spectrophotometer (UVmini‐1240, Shimadzu, Japan) against a blank. The TPC was expressed in milligrams of gallic acid equivalents per gram (mg GAE/g).

#### 2.2.3 Determination of Total Flavonoid Content

The total flavonoid content (TFC) was determined by the method described by da Silva et al. (2015). 1 mL of each sample extract was treated with aluminum trichloride (1 mL) solution in methanol (2% w/v) and 1 mL of potassium acetate solution (1 M). After 10 min of incubation at 25°C, absorbance readings were taken at 430 nm against a blank. The flavonoid content was expressed in milligrams of quercetin equivalents per gram (mg QE/g).

#### 2.2.4 DPPH Free‐Radical Scavenging Assay

The radical scavenging activity was assessed using the 2,2′‐diphenyl‐1‐picrylhydrazyl (DPPH) test as described by Rumpf et al. (2023). A sample extract (50 μL) was added to a freshly prepared methanolic DPPH solution (1950 μL). After 30 min of incubation at room temperature (25°C) in the dark, absorbance readings were taken at 517 nm. However, methanol was taken as the control, and ascorbic acid (vitamin C) was used as the standard.

#### 2.2.5 Free Radical Scavenging Using the ABTS Radical

The ABTS radical cation decolorization assay evaluated each sample extract's free radical scavenging capacity. For the assay, the ABTS+• solution was diluted in deionized water or ethanol to an absorbance of 0.7 ± 0.02 at 734 nm. A solvent blank reading was recorded (AB). After adding 100 μL of the sample extract solution (depending on solubility) to 3 mL of the ABTS+• solution, the absorbance was measured at 30°C, 10 min after initial mixing (AE).

#### 2.2.6 Ferric Reducing Antioxidant Potential (FRAP) Assay

**FIGURE 1:**
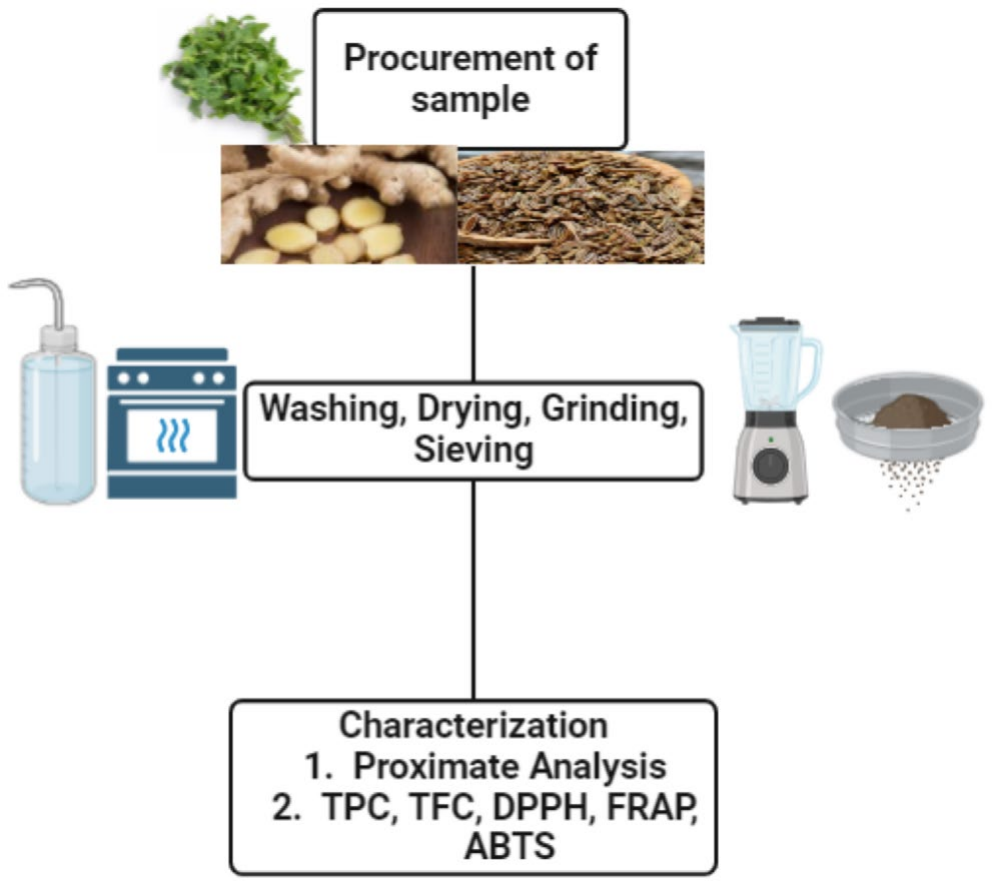
Prisma of the nutraceutical's preparation.

The ferric‐reducing antioxidant power of each sample extract was determined using a modified FRAP assay. The working FRAP reagent was prepared by mixing 10 volumes of 300 mM acetate buffer (pH 3.6) with 1 volume of 10 mM TPTZ (2,4,6‐tri(2‐pyridyl)‐s‐triazine) in 40 mM HCl and 1 volume of 20 mM ferric chloride. A standard curve was created using various concentrations of FeSO_4_ × 7H_2_O. On the day of preparation, 100 μL of sample solution and 300 μL of deionized water were added to 3 mL of freshly prepared FRAP reagent. The reaction mixture was incubated for 30 min at 37°C in a water bath, and the absorbance was measured at 593 nm. A sample blank using acetate buffer was also recorded. A flow diagram of nutraceutical preparation is presented in Figure 1.

### 2.3 Study Registration and Ethical Approval

The current study was registered on https://register.clinicaltrials.gov with identifier NCT06157034. An approval from the Institutional Review Board (IRB), Sheikh Zayed Medical College/Hospital, (258/IRB/SZMC/SZH) Rahim Yar Khan, was taken and performed according to the Helsinki Declaration. Informed consents were taken in written form from the study samples.

#### 2.3.1 Study Design and Sampling Technique

A total of 200 participants were enrolled in the study and divided into four groups (placebo, ginger, caraway, and peppermint). Out of the 200 participants, all groups (placebo; *n* = 48, ginger; *n* = 47, caraway; *n* = 48, and peppermint; *n* = 46) completed all three phases (baseline, intervention, and washout) of the study. However, 11 participants were dropped from the study. It was a randomized controlled trial. Baseline data collection marked the initiation of Phase 1 of the study. Subsequently, registered patients returned for their second dose of Anti‐Tuberculosis Treatment (ATT) after 1 month of treatment. Samples were then selected, and the intervention was administered for 3 months as part of Phase 2. Following this intervention period, Phase 3 (washout) was conducted after withholding treatment for 1 month.

**FIGURE 2:**
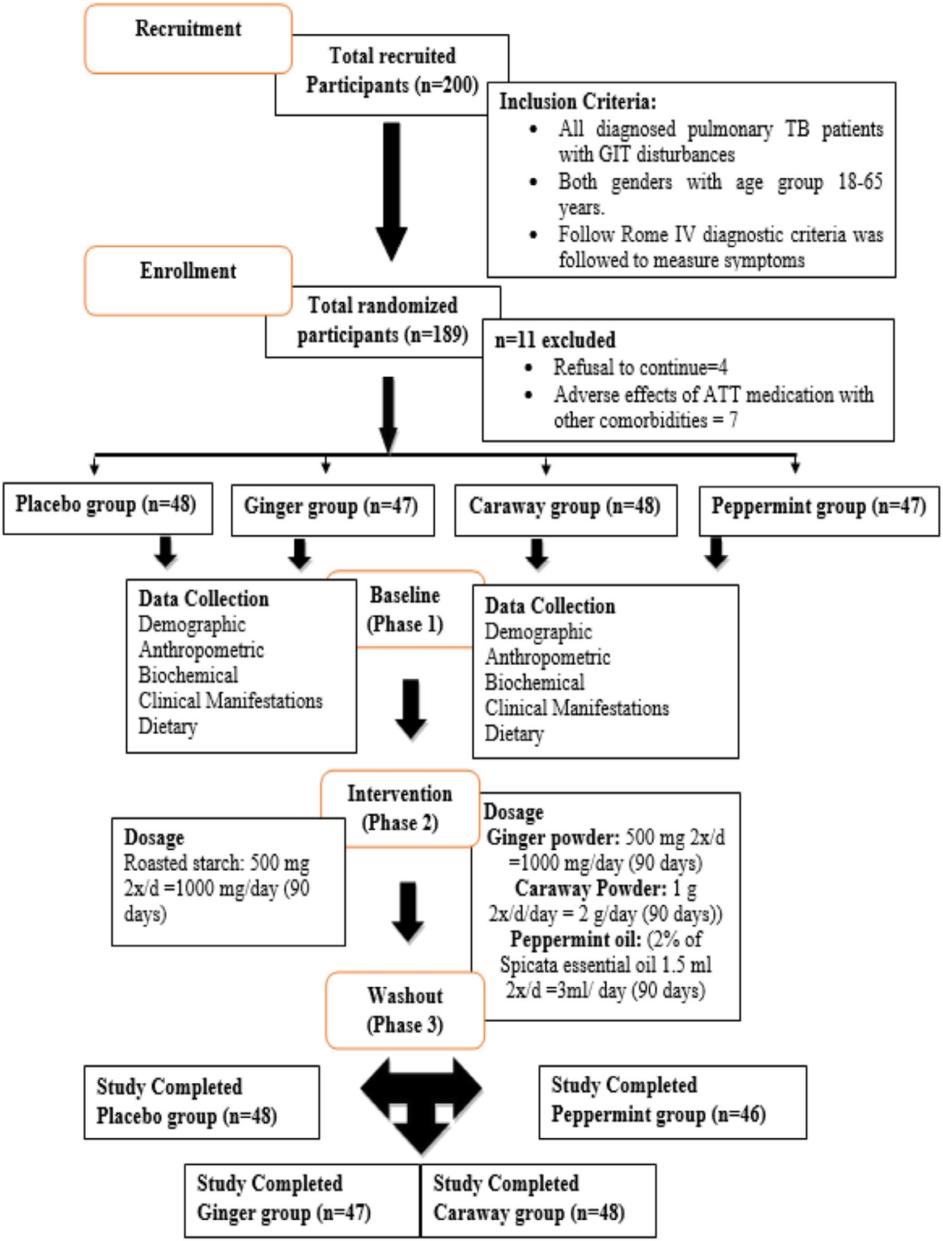
PRISMA for the recruitment of participants for the trial.

The sampling was done in two steps. In the first step, the registered population of pulmonary tuberculosis (PTB) under the National TB Program was sampled in the Pulmonology Outpatient Department (OPD), Sheikh Zayed Hospital, Rahim Yar Khan, Pakistan. Subsequently, in step 2, subjects were randomized for each treatment through systematic random sampling. The subjects were selected according to predefined criteria. The inclusion criteria were all diagnosed pulmonary TB patients with GIT disturbance symptoms from both genders aged 18–65. Rome IV diagnostic criteria were followed to measure symptoms (Palsson et al. 2016). A flow diagram of the study steps has been presented in Figure 2.

#### 2.3.2 Prescribed Doses and Blinding

Placebo group: 500 mg 2×/d = 1000 mg/day of roasted starch.

Intervention ( *Zingiber officinale* ) Group: *Zingiber officinale* powder encapsulated (500 mg 2×/d = 1000 mg/day) after breakfast and dinner (Crichton et al. 2022).

Intervention (*Caram carvi*) Group: *Carum carvi* powder encapsulated (1000 mg 2×/d = 2 g/day) after breakfast and dinner (Mahboubi 2019).

Intervention (*Mentha spicata L*.) Group: *Mentha spicata L* oil encapsulated (1.5 mL 2×/d = 3 mL/day) after breakfast and dinner (Mahboubi 2021).

All selected nutraceutical capsules were packaged in identical bottles with 2‐digit coding (11, 22, 33, *n*). The participants, caregivers, and outcome assessors were blinded to group assignments until the completion of the study. It is important to note that the ginger and placebo capsules were identical in package, size, shape, and color. However, ginger powder was added to the placebo capsule bottles to impart a ginger aroma. The intervention phase continued for 90 days (D'Cunha et al. 2018; Salekzamani et al. 2023). Patients were advised to continue their usual diet; however, they were also advised not to consume selected nutraceutical‐based products in any amount for the duration of the study. The enrolled patients completed a self‐structured questionnaire following the Rome IV diagnostic criteria for gastrointestinal disturbances to assess their symptoms. All the study participants were on ATT medications and instructed not to change their ATT medication dose during the study.

#### 2.3.3 Inclusion Criteria

- All diagnosed pulmonary TB patients have GIT disturbance symptoms.
- From both genders.
- Age group 18–65 years.
- Symptoms criteria (Rome IV) (Palsson et al. 2016).

#### 2.3.4 Exclusion Criteria

- Patients > 18 years and < 65 years.
- Patients have other chronic systemic diseases like malignancy, chronic liver disease, and chronic kidney disease.

### 2.4 Outcome Measures

The primary outcome measures were assessing the symptoms of gastrointestinal disturbances by using the Rome IV diagnostic criteria for pulmonary TB patients (Tack and Drossman 2017). The secondary measures were body weight and height, BMI, smoking, and family history of pulmonary TB. A sputum sample of each participant was also collected. Blood samples were taken from all participants after 14 h of overnight fasting to measure ESR (mL/h), WBC (cells/μL), Hb (g/dL), HCV, HIV, ALT (SGPT) (U/L), AST (SGOT) (U/L), and bilirubin (μmol/L). A 24‐h dietary record was also documented for each study participant. Additionally, all patients were instructed to report any observed adverse events during the study.

### 2.5 Statistical Analysis

All statistical analyses were performed using the Minitab software (version 20.3). The normality of the data was measured by the Shapiro–Wilk test. To compare quantitative variables between groups, an independent sample *t*‐test was employed. The chi‐squared test was applied to compare qualitative variables in both groups. Cramer's *V* test was conducted to evaluate the association between variables. Mean ± standard error (SE) signified quantitative data, while frequency and percentage represented qualitative variables. A significance level of *p* < 0.05 was adopted for all statistical tests. The hierarchical heatmap graph was designed using TBTools II.

## 3 Results and Discussion

### 3.1 Characterization of Selected Nutraceuticals

#### 3.1.1 Proximate Analysis

The proximate analysis to assess the compositional profile of ginger ( *Zingiber officinale* ) revealed significant constituents, which are presented in Table 1. Ginger showed a moisture content of 81.67%, indicating its relatively high water content. The ash content was found to be 1.03%. Subsequently, protein content was recorded at 3.11%. Crude fat content was relatively low at 5.29%, whereas crude fiber was present at 7.49%. Carbohydrates constituted a significant portion, representing 13.88%, highlighting ginger's role as a carbohydrate‐rich food source. Agu et al. (2016) studied the compositional profile of ginger and reported findings that are similar to those of the current study. Another study, Ma et al. (2021) and Ogbuewu et al. (2014) reported similar findings in this context. Chukwudi et al. (2020) also reported the compositional profile of ginger.

**TABLE 1:** Proximate analysis of selected nutraceuticals.

Nutraceuticals	Moisture (%)	Ash (%)	Protein (%)	Crude fat (%)	Crude fiber (%)	Carbohydrates (%)
Ginger (*Zingiber officinale*)	81.67 ± 3.13	1.03 ± 0.35	3.11 ± 0.52	0.29 ± 0.06	3.49 ± 0.19	13.88 ± 2.73
Caraway (*Carum carvi*)	2.97 ± 0.36	8.74 ± 0.37	29.72 ± 0.23	1.29 ± 0.07	45.11 ± 0.21	6.67 ± 0.39
Peppermint (*Mentha piperita* L.)	81.74 ± 5.52	2.71 ± 0.84	15.94 ± 0.38	54.47 ± 0.31	4.81 ± 0.14	0.79 ± 0.17

Further, Caraway seeds powder had substantially lower moisture content (2.97%) and higher ash content (8.74%), indicating a relatively concentrated composition. It was also seen that it was particularly high in protein (29.72%) and crude fiber (45.11%), suggesting that it could provide significant amounts of protein and dietary fiber in a smaller serving. The crude fat content (1.29%) was moderate, while the carbohydrate content (6.67%) was relatively low. Abdalaziz et al. (2017) reported the proximate profile of caraway that was aligned with the present study.

Additionally, the composition of peppermint oil was unique, being represented in terms of protein (15.94%), crude fat (54.47%), and crude fiber (4.81%). The moisture content of peppermint oil was measured at 81.74%, while the ash and carbohydrate content of peppermint oil were 2.71% and 0.79%, respectively. However, the composition variations highlighted the nutraceuticals' diverse nutritional profiles, indicating their potential roles in different dietary interventions. Mehri et al. (2015) and Saeed et al. (2014) described the proximate profile of peppermint oil sources as somewhat similar to the findings of the current study.

#### 3.1.2 Total Phenolic and Flavonoid Content Profile of Selected Nutraceuticals

**FIGURE 3:**
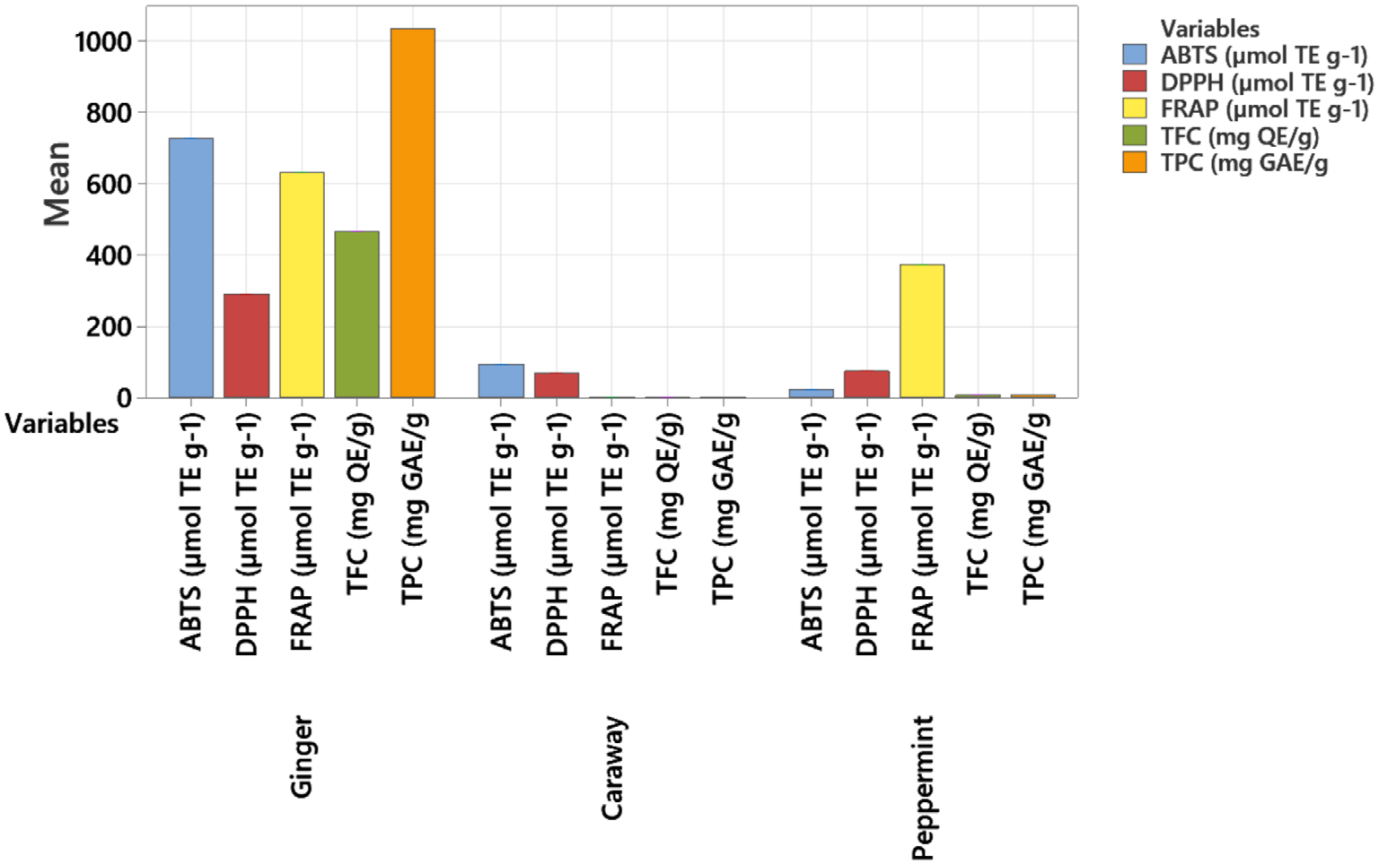
Functional properties of selected nutraceuticals.

The Total Phenolic Content (TPC) measures the concentration of phenolic compounds present in a substance, expressed in milligrams of gallic acid equivalents (mg GAE) per gram of sample. Phenolic compounds play a crucial role in the antioxidant activity of plant materials to scavenge free radicals (Siddeeg et al. 2018; Zaidi and Dahiya 2015). Figure 3 indicates that ginger ( *Zingiber officinale* ) has the highest total phenolic content (TPC) at 1035.51 mg GAE/g and total flavonoid content (TFC) at 465.34 mg QE/g among the three selected nutraceuticals. Various intrinsic and extrinsic factors, such as cultivar variations, seedling, and harvesting conditions, may be attributable to the amount of phenolic compounds (Ezez and Tefera 2021). Caraway ( *Carum carvi* ) has the lowest TPC at 1.49 mg GAE/g and TFC at 1.26 mg QE/g due to its comparatively low hydrophobicity (Vallverdú‐Queralt et al. 2015).

In addition, peppermint (*Mentha piperita L*.) fell in between, with TPC at 7.84 mg GAE/g and TFC at 6.72 mg QE/g. Zaidi and Dahiya (2015) indicated the highest total phenolic content at 12.63 ± 0.878 in Mentha piperita. However, the variation in the concentration of TPC and TFC among selected nutraceuticals could be attributed to the extraction method and solvents. High ethanolic solvent extraction has a higher dielectric constant, leading to more release of phenolic content (Tohma et al. 2017).

#### 3.1.3 Antioxidant Assay (DPPH, FRAP, and ABTS) of Selected Nutraceuticals

Figure 3 further illustrates the antioxidant capacities of three nutraceuticals, ginger ( *Zingiber officinale* ), caraway ( *Carum carvi* ), and peppermint (*Mentha piperita L*.) measured through DPPH, FRAP, and ABTS assays. Each assay offers insights into different aspects of antioxidant activity and is expressed in μmol of Trolox equivalents per gram (μmol TE g^−1^).

In the DPPH assay, antioxidants can convert the stable DPPH radical into the diphenyl‐picrylhydrazine (DPPH‐H), where a hydrogen‐donating antioxidant facilitates the reduction of DPPH, forming the non‐radical DPPH‐H (Tohma et al. 2017; Yang et al. 2018). Further, the FRAP assay can evaluate the antioxidant capacity of a substance by assessing its ability to reduce ferric ions (Fe^3+^) to ferrous ions (Fe^2+^) in the reaction medium.

Ginger exhibited high antioxidant activity across all three assays, with values for DPPH (291.47 ± 122.81 μmol TE g^−1^), FRAP (630.53 ± 275.94 μmol TE g^−1^), and ABTS (728.15 ± 267.38 μmol TE g^−1^). Our study aligned with the research reported by Tohma et al. (2017), demonstrating that ginger exhibited a significant inhibitory effect on DPPH radicals. The high antioxidant capacity can be attributed to the presence of bioactive compounds such as shogaols, gingerols, and paradols, exhibiting strong free radical scavenging abilities (Alolga et al. 2022). In contrast, caraway demonstrated much lower antioxidant activity, with DPPH (69.16 ± 0.11 μmol TE g^−1^), FRAP (1.38 ± 0.02 μmol TE g^−1^), and ABTS (95.29 ± 0.73 μmol TE g^−1^). The findings of the current study regarding FRAP activity were in close agreement with previous literature reported by Offei‐Oknye et al. (2015). Daga et al. (2022) discussed the significant antioxidant capacity of spices.

Peppermint showed moderate antioxidant activity with DPPH (73.82 ± 0.61 μmol TE g^−1^) and FRAP (373.78 ± 24.06 μmol TE g^−1^). However, it had notably low activity in the ABTS assay (21.81 ± 0.44 μmol TE g^−1^) when compared to other sources (ginger, caraway). López et al. (2010) studied the antioxidant activities of 5 Mentha species, indicating the best activity of peppermint. Meanwhile, Elmastaş et al. (2015) also reported the significant antioxidant capacity of peppermint. Wu et al. (2019) reported the effective supplementation of peppermint oil in reducing oxidative stress.

### 3.2 Study Demographics

Table 2 displays the demographics of the studied participants in alleviating gastrointestinal disturbances. The mean age of participants was 39.03 ± 18.90 years. The age distribution showed a notable gender difference. Among females, the maximum participants were 20.5% aged between 15–24 years. In contrast, 21% of males participated in the age group aged 55–65 years. Unemployment was reported by 7% of females and 22.5% of males. Daily wage jobs were held by 6% of females and 33.5% of males. Interestingly, 30% of females were housewives compared to only 2 (1%) males, highlighting a significant gender disparity in domestic roles. Furthermore, among females, none were graduates, and only 0.5% had completed matriculation, whereas 19.5% had a middle school education, 16% had primary education, and 7% were illiterate. Only 0.5% of males graduated, 21% completed matriculation, 17.5% with middle school education, 6.5% with primary education, and 11.5% were illiterate. The mean family income was Rs. 17,385 ± 528.72, and the mean BMI was 19.43 ± 5.67. A significant portion of the patients, about 55.5%, had a family history of tuberculosis, while 44.5% did not, highlighting a considerable risk factor present in over half of the participants. Smoking prevalence was exceptionally high, with 83.5% of participants, which may have implications for respiratory health and disease outcomes among the participants. However, findings indicated that age significantly influences the management and response to nutraceutical interventions due to different metabolic rates and physiological conditions across the lifespan among pulmonary TB patients (Dato et al. 2016). The findings of the current study also indicated the condition prevalent in TB patients due to the impact of the disease on appetite and nutrient absorption (Kant et al. 2015). In addition, low BMI can worsen gastrointestinal disturbances (Dumic et al. 2019). In the present study, a significant number of females were housewives, while males predominantly engaged in daily wages or were unemployed, which can influence overall health, thereby impacting gastrointestinal health among TB patients (Karl et al. 2018). Therefore, malnutrition, socio‐economic constraints, and varying levels of health literacy collectively contribute to the functional gastrointestinal disturbances among TB patients.

**TABLE 2:** Demographics of the studied participants in alleviating gastrointestinal disturbances.

Parameters		Female	Male
Age (years), mean	39.03 ± 18.90		
Age (years), *n* (%)	15–24 years	41 (20.5)	32 (16)
	25–34 years	09 (4.5)	09 (4.5)
	35–44 years	08 (4)	08 (4)
	45–54 years	09 (4.5)	23 (11.5)
	55–65 years	19 (9.5)	42 (21)
Nature of job, *n* (%)	Unemployment	14 (7)	45 (22.5)
	Daily wages	12 (6)	67 (33.5
	House wife	60 30)	2 (1)
Education, *n* (%)	Graduate	0 (0)	1 (0.5)
	Matric	1 (0.5)	42 (21)
	Middle	39 (19.5)	35 (17.5
	Primary	32 (16)	13 (6.5)
	Illiterate	14 (7)	23 (11.5)
Family income (Rs) (mean)	17,385.00 ± 528.72		
BMI Wt./Ht^2^ (mean)	19.43 ± 5.67		
Family history of TB, *n* (%)	Yes	111 (55.5)	
	No	89 (44.5)	
Smoking, *n* (%)	Yes	167 (83.5)	
	No	33 (16.5)	

### 3.3 Biochemical Analysis of Studied Participants

Table 3 depicts the contribution of sputum and biochemical analysis among the studied participants and reveals significant clinical insights into their health status. The Acid‐Fast Bacillus (AFB) results indicated that 30.5% had 1–9 AFB, 49.5% had a +1 AFB level, 16.5% had a +2 AFB level, and only 3.5% had a +3 AFB level, underlining low to moderate AFB presence in their sputum. Biochemical parameters showed a mean Erythrocyte Sedimentation Rate (ESR) of 20.38 ± 1.12 mL/h with slight inflammation. Further, the mean White Blood Cell (WBC) count was 7982.75 ± 472 cells/μL, which responded to a margin end and may reflect an immune response. The mean hemoglobin (Hb) level was 11.42 ± 0.53 g/dL, which may indicate mild anemia. However, liver enzyme: Alanine Aminotransferase (ALT/SGPT) levels were significantly elevated at 6841.25 ± 3.46 U/L, and Aspartate Aminotransferase (AST/SGOT) levels were 2135 ± 1.43 U/L, both indicating liver inflammation. Bilirubin levels averaged 3.88 ± 0.21 μmol/L. Regarding viral hepatitis markers, 5.5% tested positive for Hepatitis C virus (Anti‐HCV), 4% tested positive for Hepatitis B virus (Anti‐HBV), and 8% were positive for Hepatitis B surface antigen (HBsAg), signifying a relatively low prevalence of Hepatitis B and C.

**TABLE 3:** Sputum and biochemical analysis.

Parameters	*n* (%)
Acid fast bacillus for sputum	
1–9 AFB	61 (30.5)
+1	99 (49.5)
+2	33 (16.5)
+3	07 (3.5)
ESR (mL/h) (mean)	20.38 ± 1.12
WBC (cells/μL) (mean)	7982.75 ± 472
Hb (g/dL) (mean)	11.42 ± 0.53
ALT (SGPT) (U/L) (mean)	6841.25 ± 3.46
AST (SGOT) (U/L) (mean)	2135 ± 1.43
Bilirubin (μmol/L) (mean)	3.88 ± 0.21
Anti‐HCV	
Yes	11 (5.5)
No	189 (94.5)
Anti‐HBV	
Yes	08 (04)
No	192 (96)
HBsAg	
Yes	16 (08)
No	184 (92)

### 3.4 Assessing the Impact of Interventional Nutraceuticals With Biochemical, BMI, Calories, and Nutrient Intake in Distinct Phases of Study

Table 4 highlighted a comparison of nutraceuticals and phases in the study, with a significant association by applying an independent *t*‐test. AFB sputum, HCV, and HIV statuses remained consistently positive and negative, respectively, across all groups and phases. The ESR, an inflammation marker, showed no significant changes across groups and phases (*p* > 0.05). White blood cell (WBC) varied slightly, with higher counts observed in the ginger and caraway groups during the intervention and washout phases. Hemoglobin (Hb) levels also showed slight increases in the ginger and caraway groups during the intervention phase, with no significant association (*p* = 0.74). Moreover, liver enzymes ALT (SGPT) and AST (SGOT) showed no significant variations across groups and phases. ALT levels were slightly higher in the intervention phase, especially in the caraway group (8146 ± 4.79). Furthermore, bilirubin levels showed slight increases in ginger and caraway during the intervention phase (*p* = 0.08). Morvaridzadeh et al. (2020) reported that ginger supplementation significantly reduces the anti‐inflammatory markers that were only aligned with ESR levels of the present study. Jalali et al. (2020) also reported a significant reduction in inflammatory markers by the consumption of ginger. Further, another study described by Marlina et al. (2020) supported the reduction of the onset of breathlessness in pulmonary TB patients by aromatherapy with peppermint significantly in pulmonary TB patients.

**TABLE 4:** Comparison of interventional nutraceuticals with variables biochemical, BMI, calories, and nutrient intake in distinct phases of the study.

Parameters	Baseline				*p*	Intervention				*p*	Washout				*p*
	Placebo (*n* = 48)	Ginger (*n* = 47)	Caraway (*n* = 48)	Peppermint (*n* = 46)		Placebo (*n* = 48)	Ginger (*n* = 47)	Caraway (*n* = 48)	Peppermint (*n* = 46)		Placebo (*n* = 48)	Ginger (*n* = 47)	Caraway (*n* = 48)	Peppermint (*n* = 46)	
AFB sputum	Positive	Positive	Positive	Positive	—	Positive	Positive	Positive	Positive	—	Positive	Positive	Positive	Positive	—
HCV	Negative	Negative	Negative	Negative	—	Negative	Negative	Negative	Negative	—	Negative	Negative	Negative	Negative	—
HIV	Negative	Negative	Negative	Negative	—	Negative	Negative	Negative	Negative	—	Negative	Negative	Negative	Negative	—
ESR (mL/h)	19.73 ± 1.31	18.85 ± 1.02	21.73 ± 1.46	20.37 ± 1.23	0.47	19.69 ± 1.18	19.93 ± 1.27	22.46 ± 1.65	22.61 ± 1.35	0.32	19.71 ± 1.23	18.95 ± 1.02	24.84 ± 1.59	22.37 ± 1.77	0.21
WBC (cells/μL)	8175 ± 403	8062 ± 419	8154 ± 279	7941 ± 352	0.14	8364 ± 474	9267 ± 445	9471 ± 239	7944 ± 352	0.41	8437 ± 454	9279 ± 479	9758 ± 259	9947 ± 352	0.09
Hb (g/dL)	11.32 ± 0.29	11.36 ± 0.10	11.23 ± 0.13	11.53 ± 0.15	0.25	11.77 ± 0.17	12.41 ± 0.12	14.27 ± 0.23	11.96 ± 0.15	0.74	11.67 ± 0.29	11.28 ± 0.15	12.63 ± 0.16	13.58 ± 0.23	0.11
ALT (SGPT) (U/L)	7215 ± 3.91	7329 ± 4.09	7142 ± 4.39	6929 ± 4.06	0.46	7453 ± 4.52	7729 ± 4.09	8146 ± 4.79	7099 ± 4.56	0.93	7307 ± 4.47	8327 ± 4.59	6142 ± 4.37	6825 ± 4.56	0.12
AST (SGOT) (U/L)	2169 ± 3.94	2157 ± 5.21	2136 ± 4.89	2144 ± 5.17	0.59	2094 ± 3.53	2156 ± 5.21	2149 ± 4.54	2167 ± 5.16	0.13	2138 ± 4.45	2172 ± 5.17	2152 ± 4.49	2163 ± 4.17	0.07
Bilirubin (μmol/L)	3.83 ± 0.05	3.90 ± 0.02	3.92 ± 0.01	3.86 ± 0.09	0.27	3.77 ± 0.19	3.95 ± 0.52	4.22 ± 0.51	3.93 ± 0.57	0.08	3.78 ± 0.36	3.99 ± 0.02	3.99 ± 0.03	3.92 ± 0.07	0.11
BMI	19.43 ± 1.11	19.39 ± 1.43	19.47 ± 1.69	19.43 ± 1.58	0.17	19.59 ± 2.15	19.97 ± 1.80	19.83 ± 1.46	19.65 ± 1.66	0.24	19.78 ± 2.01	20.15 ± 1.29	19.89 ± 1.53	19.77 ± 1.96	**0.02**
Energy consumption (kcal)	1090.83 ± 136	1093.76 ± 271	1095.39 ± 159	1094.55 ± 178	**0.02**	1085.74 ± 364	1095.45 ± 357	1099.22 ± 151	1102.26 ± 195	0.19	1089.48 ± 247	1104.52 ± 262	1099.59 ± 356	1096.29 ± 401	0.18
CHO (g)	190.89 ± 2.47	191.41 ± 1.72	191.69 ± 2.03	191.54 ± 2.31	**0.04**	190.00 ± 2.73	191.70 ± 2.41	192.36 ± 1.94	192.85 ± 1.70	**0.05**	190.66 ± 3.11	193.29 ± 1.59	192.42 ± 2.74	191.85 ± 2.96	**0.02**
Protein (g)	40.90 ± 2.27	41.02 ± 1.13	41.07 ± 2.49	41.04 ± 1.21	0.13	40.72 ± 2.17	41.07 ± 1.67	41.22 ± 2.36	41.33 ± 1.89	0.26	40.86 ± 2.61	41.41 ± 2.04	41.23 ± 2.17	41.11 ± 2.33	0.30
Fat (g)	18.18 ± 1.15	18.23 ± 1.63	18.25 ± 1.77	18.24 ± 1.51	0.06	18.09 ± 1.87	18.25 ± 1.61	18.32 ± 1.49	18.37 ± 1.56	0.31	18.16 ± 1.58	18.40 ± 1.44	18.32 ± 2.09	18.27 ± 1.97	0.24
Fiber (g)	15.27 ± 1.21	15.31 ± 1.09	15.33 ± 1.17	15.32 ± 1.31	**0.05**	15.20 ± 1.35	15.33 ± 1.28	15.39 ± 1.27	15.43 ± 1.31	0.08	15.25 ± 1.23	15.46 ± 1.39	15.39 ± 1.32	15.34 ± 1.35	**0.05**

*Note*: Significance level = p < 0.05.

Abbreviations: BMI, body mass index; CHO, carbohydrates, independent *t*‐test was applied.

Body mass index remained relatively stable across all phases, with a notable but uncertain increase in the Ginger group during the washout phase (20.15 ± 1.29) might be due to the potential long‐term impact of Ginger on BMI. The significant BMI parameter was in close agreement with the study reported by Pajanivel et al. (2022), who reported improved BMI, protein, and albumin levels with a positive impact on nutritional status and quality of life among pulmonary TB patients. Moreover, the present study was conducted on patients who belong to a low social income status, which was linked to the non‐provision of enough food. Due to a shortage of sufficient food, the BMI status, as well as energy consumption, along with nutrient intake, may affect the pulmonary TB patients. This severe condition ultimately led to significant weight loss, making it challenging to observe rapid BMI improvements. Lee et al. (2020) supported culturally relevant foods for effective nutritional support.

Interestingly, energy consumption showed a significant increase at baseline (*p* = 0.02), indicating initial differences among groups. The placebo group had 1090.83 ± 136 kcal, while Ginger had 1093.76 ± 271 kcal. Though during the intervention and washout phases, energy intake improved slightly across all groups, a statistically nonsignificant (*p* = 0.19 and *p* = 0.18, respectively) association was seen. Further, carbohydrate (CHO) intake was significantly different at all phases; at baseline, the placebo group consumed 190.89 ± 2.47 g, Ginger had 191.41 ± 1.72 g (*p* = 0.04), while during the intervention and washout phases, significant differences were also observed (intervention *p* = 0.05, washout *p* = 0.02 respectively), suggesting consistent differences in carbohydrate intake among groups throughout the study. However, protein and fat intake showed no significant changes across groups and phases, with *p*‐values consistently > 0.05. Protein intake at baseline was noticed as 40.90 ± 2.27 g in Placebo, and 41.02 ± 1.13 g in Ginger (*p* = 0.13). Similarly, fat intake at baseline was 18.18 ± 1.15 g in the Placebo and 18.23 ± 1.63 g in Ginger (*p* = 0.06). Fiber intake showed some significant differences at baseline and washout phases (baseline and washout *p* = 0.05). At baseline, Placebo had 15.27 ± 1.21 g, Ginger had 15.31 ± 1.09 g, and during the washout phase, Ginger increased to 15.46 ± 1.39 g, respectively.

### 3.5 Assessing the Severity of Gastrointestinal Disturbances in Different Phases of Nutraceutical Administration

**FIGURE 4:**
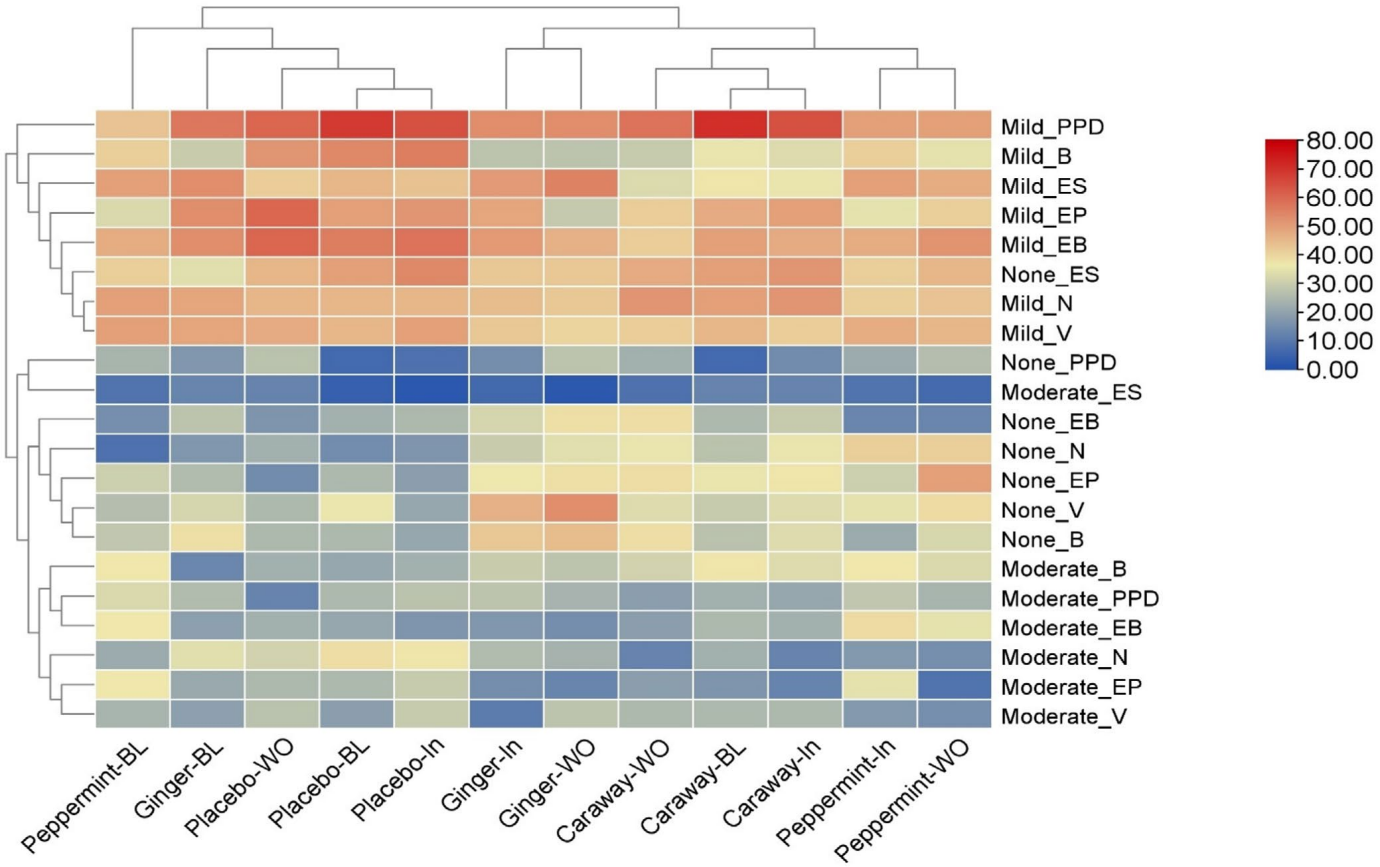
Heatmap showing intensities of gastrointestinal disturbances of placebo, ginger, caraway, and peppermint interventions during different phases of the study. B, Belching; BL, Baseline; EB, Epigastric Burning; EP, Epigastric Pain; ES, Early Satiation; In, Intervention; N, Nausea; PPD, Postprandial Distress; V, Vomiting; WO, Washout.

Figure 4 and Table S1 compared the severity levels of gastrointestinal disturbances across different treatment groups (Placebo, Ginger, Caraway, Peppermint) and phases (Baseline, Intervention, Washout). The gastrointestinal disturbances are coded as Postprandial Distress (PPD), Early Satiation (ES), Epigastric Pain (EP), Epigastric Burning (EB), Nausea (N), Vomiting (V), and Belching (B) in describing the hierarchical heatmap display (Figure 4). The heatmap categorized gastrointestinal disturbances with their intensities (Moderate, Mild, and None) across phases. The scale from 0 to 80 depicts percentages (%) of intensities that were read from red to blue. From Figure 4, it is obvious that the participants from the placebo and Caraway groups at baseline had mild PPD. Further, during the intervention phase, similar levels of gastrointestinal disturbances in the Placebo and Caraway groups were recorded with mild intensity. However, during the washout phase, patients of the Placebo group with mild EP and EB had complaints. Moreover, the respondents with no ES gastrointestinal disturbance were noted in both the Placebo Baseline and Intervention groups. Interestingly, during all phases of the study, the patients had negligible responses of moderate intensity of gastrointestinal disturbances with nutraceutical intervention. Moreover, during the washout phase, the peppermint group showed no EP complaints. Peppermint oil showed promising effects in pediatric IBS (Kline et al. 2001) as peppermint oil is well tolerated in children (Fifi et al. 2018). Further, Madisch et al. (2023) reported the reduction in GI disturbances with the consumption of peppermint and caraway oil.

The ginger group in both intervention and washout phases showed no vomiting recurrence. A reduction in vomiting episodes was reported by Smith et al. (2004) with the consumption of functional food. In addition, the ginger group during the washout phase was reported to lower the intensity of PPD and ES among patients, as shown in Figure 4. Attari et al. (2019) confirmed a significant association between lowering the intensity of functional gastrointestinal disturbance with ginger consumption, which was also observed in the present study. Chumpitazi et al. (2018) reported that ginger reduces the subjective experience of pain and nausea in some proinflammatory conditions among patients. Yuki et al. (2015) found improvements in abdominal pain and bloating when utilizing a mixture of herbs and spices.

## 4 Conclusion

Recently, ATT has become more difficult as a result of adverse drug reactions (ADRs) that raise healthcare expenses and negatively impact patients' quality of life. Therapeutic herbal interventions, specifically ginger, caraway, and peppermint, showed promising potential in alleviating GI disturbances such as nausea, vomiting, bloating, and abdominal pain in PTB patients. Incorporating herbal components as complementary treatments with medications improves TB patients' overall quality of life. This study has emphasized the need for healthcare providers to consider individualized treatment plans that integrate these herbal options, ultimately leading to better patient outcomes and satisfaction.

## Author Contributions

**Asma Latif:** conceptualization (equal), formal analysis (equal), methodology (equal), writing – original draft (equal), writing – review and editing (equal). **Hajra Ahmad:** conceptualization (equal), methodology (equal), supervision (equal). **Imran Bashir:** resources (equal).

## Conflicts of Interest

The authors declare no conflicts of interest.

## Supporting information


Table S1

## Data Availability

Data will be provided on request.
