# Supplementary material for: Comparative Study on the Prokinetic Effects of Ginger, Caraway, and Peppermint in Relieving Gastrointestinal Disturbances in Pulmonary TB Patients: A Clinical Trial
Source: Food Sci Nutr. 2025 May 25;13(6):e70338. doi: 10.1002/fsn3.70338 (PMC12104075; doi:10.1002/fsn3.70338)
Supplement: Supplementary file 1 — Table S1 [file FSN3-13-e70338-s001.docx]

**Supplementary Table 1: Alleviating the intensity of Gastrointestinal disturbances by nutraceutical intervention during different phases of the study**

| Gastrointestinal disturbances | Intensity | Baseline | | | | Cramer’s V | Intervention | | | | Cramer’s V | Washout | | | | Cramer’s V | |
| --- | --- | --- | --- | --- | --- | --- | --- | --- | --- | --- | --- | --- | --- | --- | --- | --- | --- |
|  |  | **Placebo**  **(n=48)**  **n (%)** | **Ginger**  **(n=47)**  **n (%)** | **Caraway**  **(n=48)**  **n (%)** | **Peppermint**  **(n=46) n (%)** |  | **Placebo**  **(n=48)**  **n (%)** | **Ginger**  **(n=47)**  **n (%)** | **Caraway**  **(n=48)**  **n (%)** | **Peppermint**  **(n=46)**  **n (%)** |  | **Placebo**  **(n=48)**  **n (%)** | **Ginger**  **(n=47)**  **n (%)** | **Caraway**  **(n=48)**  **n (%)** | **Peppermint**  **(n=46) n (%)** |  |  |
| Post prandial distress | **None** | 03 (6.25) | 08 (17.02) | 03 (6.25) | 11 (23.91) | 0.437 | 04 (8.33) | 07 (14.89) | 07 (14.5) | 10 (21.74) | 0.352 | 13 (27.08) | 13 (27.66) | 11 (22.91) | 12 (26.09) | 0.204 |  |
|  | **Mild** | 33 (68.75) | 27 (57.45) | 34 (70.83) | 20 (43.48) |  | 31 (64.58) | 26 (53.32) | 31 (64.58) | 23 (50) |  | 29 (60.42) | 25 (53.19) | 28 (58.33) | 23 (50) |  |  |
|  | **Moderate** | 12 (25) | 12 (25.53) | 11 (22.91) | 15 (32.61) |  | 13 (27.08) | 13 (27.66) | 10 (20.83) | 13 (28.26) |  | 06 (12.5) | 12 (25.53) | 09 (18.75) | 11 (23.91) |  |  |
| Early Satiation | **None** | 24 (50) | 16 (34.02) | 24 (50) | 19 (41.30) | 0.482 | 26 (54.17) | 20 (42.55) | 25 (52.08) | 19 (41.30) | 0.475 | 22 (45.83) | 20 (42.55) | 23 (47.91) | 21 (45.65) | 0.427 |  |
|  | **Mild** | 22 (45.83) | 25 (53.19) | 18 (37.5) | 23 (50) |  | 21 (43.75) | 24 (51.06) | 17 (35.41) | 23 (50) |  | 20 (41.67) | 26 (55.32) | 21 (43.75) | 22 (47.83) |  |  |
|  | **Moderate** | 02 (4.16) | 06 (12.77) | 06 (12.50) | 04 (8.70) |  | 01 (2.08) | 03 (6.38) | 06 (12.5) | 04 (8.70) |  | 06 (12.5) | 01 (2.13) | 04 (8.33) | 03 (6.52) |  |  |
| Epigastric Pain | **None** | 12 (25) | 12 (25.53) | 17 (35.41) | 14 (30.43) | 0.063 | 09 (18.75) | 17 (36.17) | 18 (37.5) | 14 (30.43) | 0.126 | 07 (14.5) | 18 (38.30) | 19 (38.58) | 23 (50) | 0.119 |  |
|  | **Mild** | 24 (50) | 25 (53.19) | 23 (47.91) | 15 (32.61) |  | 25 (52.08) | 23 (48.94) | 24 (50) | 16 (34.78) |  | 29 (60.42) | 23 (48.94) | 20 (41.67) | 19 (41.30) |  |  |
|  | **Moderate** | 12 (25) | 10 (21.28) | 08 (16.67) | 17 (36.96) |  | 14 (29.16) | 07 (14.89) | 06 (12.5) | 16 (34.78) |  | 12 (25) | 06 (12.77) | 09 (18.75) | 04 (8.70) |  |  |
| Epigastric Burning | **None** | 11 (22.91) | 13 (27.66) | 12 (25) | 07 (15.22) | 0.019 | 12 (25) | 15 (31.91) | 14 (29.16) | 06 (13.04) | 0.149 | 08 (16.67) | 18 (38.30) | 19 (38.58) | 06 (13.04) | 0.066 |  |
|  | **Mild** | 27 (56.25) | 25 (53.19) | 24 (50) | 22 (47.83) |  | 28 (58.33) | 24 (51.06) | 23 (47.91) | 22 (47.83) |  | 29 (60.42) | 22 (46.81) | 20 (41.67) | 24 (52.17) |  |  |
|  | **Moderate** | 10 (20.83) | 09 (19.15) | 12 (25) | 17 (36.96) |  | 08 (16.67) | 08 (17.02) | 11 (22.91) | 18 (39.13) |  | 11 (22.91) | 07 (14.89) | 09 (18.75) | 16 (34.78) |  |  |
| Nausea | **None** | 07 (14.58) | 08 (17.02) | 13 (27.08) | 13 (28.26) | 0.646 | 08 (16.67) | 14 (29.79) | 17 (35.41) | 19 (41.30) | 0.553 | 11 (22.91) | 16 (34.02) | 17 (35.41) | 19 (41.30) | 0.740 |  |
|  | **Mild** | 22 (45.83) | 23 (48.94) | 24 (50) | 23 (50) |  | 22 (45.83) | 21 (44.68) | 25 (52.08) | 19 (41.30) |  | 22 (45.83) | 20 (42.55) | 25 (52.08) | 20 (43.48) |  |  |
|  | **Moderate** | 19 (38.58) | 16 (34.02) | 11 (22.91) | 10 (21.74) |  | 18 (37.5) | 12 (25.53) | 06 (12.5) | 08 (17.39) |  | 15 (31.25) | 11 (23.40) | 06 (12.5) | 07 (15.22) |  |  |
| Vomiting | **None** | 17 (35.71) | 15 (31.91) | 14 (29.16) | 12 (26.09) | 0.721 | 10 (20.83) | 22 (46.81) | 16 (33.33) | 16 (34.78) | 0.613 | 12 (25) | 25 (53.19) | 16 (33.33) | 18 (39.13) | 0.683 |  |
|  | **Mild** | 22 (45.83) | 23 (48.94) | 22 (45.83) | 23 (50) |  | 24 (50) | 20 (42.55) | 20 (41.67) | 22 (47.83) |  | 23 (47.91) | 19 (40.43) | 20 (41.67) | 21 (45.65) |  |  |
|  | **Moderate** | 09 (18.75) | 09 (19.15) | 12 (25) | 11 (23.91) |  | 14 (29.16) | 05 (10.64) | 12 (25) | 08 (17.39) |  | 13 (27.08) | 13 (27.66) | 12 (25) | 07 (15.22) |  |  |
| Belching | **None** | 12 (25) | 18 (38.30) | 13 (27.08) | 13 (28.26) | 0.868 | 10 (20.83) | 20 (42.55) | 16 (33.33) | 10 (21.74) | 0.790 | 12 (25) | 21 (44.68) | 19 (38.58) | 15 (32.61) | 0.694 |  |
|  | **Mild** | 26 (54.17) | 14 (29.79) | 17 (35.41) | 19 (41.30) |  | 27 (56.25) | 13 (27.66) | 16 (33.33) | 19 (41.30) |  | 25 (52.08) | 13 (27.66) | 14 (29.16) | 16 (34.78) |  |  |
|  | **Moderate** | 10 (20.83) | 15 (31.91) | 18 (37.5) | 17 (36.96) |  | 11 (22.91) | 14 (29.79) | 16 (33.33) | 17 (36.96) |  | 11 (22.91) | 13 (27.66) | 15 (31.25) | 15 (32.61) |  |  |

**Cramer’s V test (Weak association=0.1, Medium association=0.4, Large association=0.5)**
